# Arsenic exposure and risk of preeclampsia in a Mexican mestizo population

**DOI:** 10.1186/s12884-016-0946-4

**Published:** 2016-07-11

**Authors:** Ada Sandoval-Carrillo, Edna M. Méndez-Hernández, Elizabeth I. Antuna-Salcido, Sergio M. Salas-Pacheco, Fernando Vázquez-Alaniz, Alfredo Téllez-Valencia, Marisela Aguilar-Durán, Marcelo Barraza-Salas, Francisco X. Castellanos-Juárez, Osmel La Llave-León, José M. Salas-Pacheco

**Affiliations:** Institute of Scientific Research, Juarez University of the State of Durango, Av. Universidad y Fanny Anitua s/n. Col. Centro, C.P. 34000 Durango, Dgo Mexico; Faculty of Medicine and Nutrition, Juarez University of the State of Durango, Zip Code 34000 Durango, Mexico; General Hospital 450, Health Services, Durango, Zip Code 34000 Mexico; Faculty of Chemical Sciences, Juarez University of the State of Durango, Zip Code 34000 Durango, Mexico

**Keywords:** Preeclampsia, Arsenic, Drinking water

## Abstract

**Background:**

Exposure to arsenic in drinking water has been associated with various complications of pregnancy including fetal loss, low birth weight, anemia, gestational diabetes and spontaneous abortion. However, to date, there are no studies evaluating its possible association with preeclampsia.

**Methods:**

This case–control study involved 104 preeclamptic and 202 healthy pregnant women. The concentrations of arsenic in drinking water and urine were measured using a Microwave Plasma-Atomic Emission Spectrometer.

**Results:**

We found relatively low levels of arsenic in household tap water (range of 2.48–76.02 μg/L) and in the urine of the participants (7.1 μg/L vs 6.78 μg/L in cases and controls, respectively).

**Conclusions:**

The analysis between groups showed for the first time that at these lower levels of exposure there is no association with preeclampsia.

## Background

Preeclampsia (PE) is a disorder peculiar to pregnancy and a major cause of maternal death and adverse fetal outcome [1]. In developing countries where access to health care is limited, PE is a leading cause of maternal mortality, with estimates of more than 60,000 maternal deaths per year [2] Although the exact pathophysiologic mechanisms of PE remain elusive, studies to date have implicated multiple processes, including the following: abnormal trophoblastic invasion, vasospasm, platelet activation, imbalance in the vasomotor-regulating factors and placental ischemia [3]. PE is characterized by increased oxidative stress due to the imbalance between lipid peroxidation and antioxidant defense mechanisms, leading to endothelial dysfunction and free radical mediated cell injury [4].

Arsenic-contaminated drinking water represents a major public health problem internationally [5–8]. The World Health Organization (WHO) and U.S. Environmental Protection Agency (EPA) standard for arsenic level in drinking water is 10 μg/L [9, 10]. Arsenic (As) is an established carcinogen and is also associated with a wide range of other chronic illnesses, such as diabetes, hypertension, and vascular diseases [11].

Oxidative stress has been identified as an important mechanism of As toxicity and carcinogenicity. In particular, As induces oxidative DNA damage and lipid peroxidation [12–16]. Oxidative stress and disrupted antioxidant systems have been shown to be involved in a wide range of pregnancy complications such as impaired fetal growth, PE, and miscarriage [17, 18].

Besides the generation of oxidative stress as a possible mechanism by which As may be associated with PE, Shin Le et al. reported that exposure to environmentally relevant concentrations of As (2.5 μM of AsNaO2) inhibit the migration of EVT cells (a human extravillous trophoblast cell line) in vitro, therefore, a similar mechanism may be occurring in vivo [19].

Several studies have been conducted to determine the association between chronic As exposure and adverse pregnancy outcome. Excess spontaneous abortion, stillbirth, and preterm birth rates among women with chronic As exposure have been reported [20–23]. However, to date there are no reports that show an association between As exposure and PE. This study evaluates whether As exposure from drinking water is associated with PE in a population of northern Mexico.

## Methods

### Patient recruitment

This prospective case–control study was approved by the Research Ethics Committee of the General Hospital of the Ministry of Health of Durango, Mexico in accordance with the Code of Ethics of the Declaration of Helsinki. Signed informed consent was obtained from all patients and controls before participation in the study. The sample size was calculated using the formula *n* = (Z_α/2_ + Z_β_)^2^ ṗ (1–ṗ) (r + 1)/d^2^r. The n needed to achieve 80 % power with an alpha of 0.05 was 94 (cases) and 188 (controls). Finally, we recruited 104 women diagnosed with PE (cases) and 202 healthy pregnant women (controls). The inclusion criteria were all those women diagnosed with mild PE (blood pressure (BP) ≥ 140/90 mmHg and proteinuria ≥ 30 mg/dL), severe PE (BP ≥ 160/110 mmHg and proteinuria ≥ 2000 mg/dL) and eclampsia (defined as occurrence, in a woman with PE, of seizures that cannot be attributed to other causes). The control group was conformed by healthy pregnant women attending the same hospital; without hypertensive, pathological or metabolic disorders during pregnancy. Follow up was given to the control group to corroborate the normality of the blood pressure values.

### Sample collection

Within 1–3 weeks of delivery, a drinking water sample was collected at the homes of each of the study participants. Drinking-water samples were collected based on the subject’s primary drinking water source. Maternal spot urine samples were collected at the hospital before delivery and immediately transported to the laboratory. Samples were stored at −80 °C until processing.

### Detection of As in drinking water and urine

The concentrations of As in drinking water (DW) and urine were measured in the toxicology laboratory of Scientific Research Institute of the Universidad Juárez del Estado de Durango (UJED) using a Microwave Plasma-Atomic Emission Spectrometer (MP-AES 4100). The Trace Elements in Water standard reference material (SRM 1643e) (National Institute of Standards and Technology, Gaithersburg, MD) was used for quality control. The limit of detection for As in DW by MP-AES was 0.5 μg As/L. For urine analysis, six point calibration curves were prepared. To compensate for variation in the dilution of the urine (caused by variation in fluid intake, time of sampling, temperature, and physical activity), we adjusted the concentrations by specific gravity.

### Statistical analysis

Independent sample Student’s *t*-tests were performed using SPSS software (version 15.0; SPSS Inc., Chicago, IL, USA). Odds ratios (ORs) as estimates of relative risk of the disease were calculated with 95 % confidence intervals (95 % CIs). The ORs were adjusted for variations in age and weeks of pregnancy by means of a multivariate logistic regression model. Mann–Whitney *U* test was used when the data were not normally distributed. For analysis, our patients were stratified into 3 groups based on As levels in DW (Table 3). The Group 1 (G1) presented levels lower than 10 μg/L, group 2 (G2) levels between 10.1 μg/L and 25 μg/L and group 3 (G3) levels above 25.1 μg/L

## Results

Clinical characteristics for controls and cases are shown in Table 1. Of the 104 women diagnosed with PE, 13 had mild PE, 72 severe PE and 19 eclampsia. Variables that showed a difference between groups were family history of PE, systolic and diastolic blood pressure (mm Hg), weeks of pregnancy and body mass index (Table 1). The range of As concentration in household tap water was 2.48–76.02 μg/L with more than 95 % of the participants having As levels higher than 10 μg/L. The mean concentration of As in DW was 39.58 μg/L and 40.49 μg/L for cases and controls, respectively; there were no statistically significant differences (Table 2, *p* = 0.816). While the WHO sets a maximum concentration of 10 μg/L in DW, the authorities in Mexico have set a maximum concentration of 25 μg/L (NOM-127-SSA1-1994) [24]. For this reason, the OR was estimated stratifying our patients into 3 groups based on As levels in DW. The results of Table 3 show that although the group exposed to concentrations above 25 μg/L presents an increased risk (OR = 1,715). This difference is not statistically significant (*p* = 0.214).

**Table 1:** Clinical characteristics for cases and controls

Clinical features	Controls (*n = 202*)	Cases (*n = 104*)	P-*value*
Age (years)	24.30 (7.078)^a^	24.39 (7.349)^a^	.92^b^
Weeks of pregnancy	37.49 (3.96)^a^	35.82 (3.97)^a^	0.001^b^
Systolic BP (mm Hg)	111.74 (10.82)^a^	158.36 (16.41)^a^	<0.0001^b^
Diastolic BP (mm Hg)	70.39 (9.97)^a^	101.21 (10.3)^a^	<0.0001^b^
Number of pregnancies	2.26 (1.40)^a^	2.34 (2.49)^a^	0.718^b^
Body mass index	24.61 (5.22)^a^	27.63 (5.82)^a^	<0.0001^b^
PE antecedent	13/202	14/104	0.045^c^

^a^Mean ± Standard deviation

^b^Independent sample *T* test

^c^Chi square test

**Table 2:** Water and urine arsenic levels in cases and controls

Arsenic μg/L	Controls (*n = 202*)	Cases (*n = 104*)		P-*value*
Water	40.49 (16.40)^a^	39.58 (26.43)^a^		0.816^b^
Urine	6.78 (3.48)^a^	7.1 (5.74)^a^		0.428^c^
		Mild PE *n* = 13	Severe PE/eclampsia *n* = 91	P-*value*
Water		46.03 (20.65)^a^	38.62 (26.87)^a^	0.519^b^
Urine		7.82 (6.87)^a^	7.03 (5.67)^a^	0.788^c^

^a^ Mean ± Standard deviation

^b^ Independent sample *T* test

^c^ Mann–Whitney *U* test

**Table 3:** Odds ratio estimation by ranges of arsenic in water and urine

Water arsenic	OR* (95 % CI)	P-*value*	Urine arsenic	OR* (95 % CI)	P-*value*
Group 1^a^ *n = 10*	Reference		Tertile 1^d^ *n = 102*	Reference	
Group 2^b^ *n = 69*	1.486 (0.200–11.025)	0.698	Tertile 2^e^ *n = 102*	1.400 (0.748–2.621)	0.698
Group 3^c^ *n = 227*	1.715 (0.732–4.019)	0.214	Tertile 3^f^ *n = 102*	0.788 (0.411–1.512)	0.214

^a^ DW As < 10 μg/L

^b^ DW As 10.1–25 μg/L

^c^ DW As >25 μg/L

^d^ U-tAs ≤7.4956 μg/L

^e^ U-tAs >7.4956 ≤ 11.4911 μg/L

^f^ U-tAs >11.4911 μg/L

* ORs were adjusted for age and weeks of pregnancy

Total urinary As concentration (U-tAs) was also evaluated. The mean concentration of U-tAs was 7.1 μg/L and 6.78 μg/L for cases and controls, respectively; there were no statistically significant differences (Table 2, *p* = 0.428). With the intention to establish whether As may be associated with the severity of PE, the cases were stratified in mild PE and severe PE/eclampsia. The results of Table 2 show that there is no statistically significant differences in the U-tAs (*p* =0.788). The risk of PE by U-tAs was estimated piling up to the patients in tertiles. The results in Table 3 show that at these levels, U-tAs is not a risk for PE.

Finally, we evaluated the correlation between As in DW and U-tAs. We observed an increase in the U-tAs associated with higher levels of As in DW. G1 presented a mean of 3.39 μg/L, G2 of 6.67 μg/L and G3 of 7.8 μg/L. However, the correlation coefficient was very low (R^2^ = 0.036).

## Discussion

To our knowledge this is the first study that evaluates if As exposure from DW is associated with PE. The As concentrations in household tap water (2.48–76.02 μg/L) were consistent with those previously found by our working group in the wells that provide DW to the city of Durango [25, 26]. Although these concentrations are not as high as those reported in other countries [27–30] or even in other regions of our own locality [31], there is a tremendous interest in the evaluation of regions with low or moderate As exposure in accordance with the increasingly clear evidence that relatively low levels of As can have health effects. Our comparative analysis between controls and cases evidenced no statistically significant differences. In addition, no differences were found in the analysis based on the severity of the PE.

The analysis of U-tAs showed a mean of 7.1 μg/L for cases and 6.78 μg/L for controls. These U-tAs levels are clearly lower than those reported among pregnant women in Bangladesh (80 μg/L) [32] and even lower than those reported in pregnant women in the nearby region known as Comarca Lagunera (23.3 μg/L) [33]. In our study we didn’t find an association between U-tAs and PE or an association with the severity of PE. Recently, Joy-Mendez et al. found no association between serum As levels and blood pressure in a cohort of pregnant women from Mexico city [34]. They reported a mean of 15.2 μg/L of As in serum. Although they don’t evaluate PE, our results can be considered similar.

In contrast to our results, several reports have associated As exposure with pregnancy complications including low weight of the newborn [35], fetal death [36], gestational diabetes [32], anemia [37] and spontaneous abortions [38], however, these associations appear at significantly higher levels of As (e. g., fetal death, U-tAs >200 μg/L or spontaneous abortions, As in DW >100 μg/L).

Our results could be interpreted on the one hand, as a confirmation of no association between As and PE, at least at these low levels. On the other hand, they might suggest that we need higher levels of As exposure to be able to observe the association.

Our study has some limitations. Although the participants state that their main source of water is from the tap, we can’t rule out that As can come from other sources of drinking water (e.g., bottled water), some food, or by some occupational exposure. Another limitation is that we didn’t find high levels of U-tAs, so we can’t establish in our study if higher levels of urinary As are or are not associated with PE.

The evaluation of pregnant women with higher levels of As as well as the analysis of other factors (e.g., genetic or nutritional) becomes necessary to confirm and strengthen our findings.

## Conclusions

First, it is shown that the majority of our population is exposed to As levels higher than that established by the WHO. In addition, our work suggests for the first time that there is no association between As exposure and PE.

## Abbreviations

As, arsenic; DW, drinking water; EPA, environmental protection agency; ORs, odds ratios; PE, preeclampsia; UJED, Universidad Juárez del Estado de Durango; U-tAs, urinary arsenic concentration; WHO, World Health Organization
